# Penetration of Microplastics and Nanoparticles Through Skin: Effects of Size, Shape, and Surface Chemistry

**DOI:** 10.3390/jox15010006

**Published:** 2024-12-31

**Authors:** Arianna Menichetti, Dario Mordini, Marco Montalti

**Affiliations:** 1 Department of Chemistry “Giacomo Ciamician”, University of Bologna, Via Selmi 2, 40126 Bologna, Italy; arianna.menichetti2@unibo.it (A.M.); dario.mordini2@unibo.it (D.M.); 2 Department of Chemistry “Giacomo Ciamician”, University of Bologna, Tecnopolo di Rimini, Via Dario Campana, 71, 47922 Rimini, Italy

**Keywords:** microplastics, nanoplastics, nanoparticles, toxicity, drug delivery

## Abstract

Skin represents an effective barrier against the penetration of external agents into the human body. Nevertheless, recent research has shown that small particles, especially in the nanosized range, can not only penetrate through the skin but also work as vectors to transport active molecules such as contrast agents or drugs. This knowledge has opened new perspectives on nanomedicine and controlled drug delivery. On the other hand, micro- and nanoplastics represent a form of emerging pollutants, and their concentration in the environment has been reported to drastically increase in the last years. The possible penetration of these particles through the skin has become a major concern for human health. If the actual primary toxicity of these materials is still debated, their possible role in the transport of toxic molecules through the skin, originating as secondary toxicity, is surely alarming. In this review paper, we analyze and critically discuss the most recent scientific publications to underline how these two processes, (i) the controlled delivery of bioactive molecules by micro- and nano-structures and (ii) the unwanted and uncontrolled penetration of toxic species through the skin mediated by micro- and nanoparticles, are deeply related and their efficiency is strongly affected by the nature, size, and shape of the particles.

## 1. Overview

This review presents a novel comparative approach, focusing on the exploration of similarities between two seemingly disparate research areas: the transdermal delivery of drugs via nanoparticles and the cutaneous absorption of microplastics. The aim is to highlight the existing connections between these two phenomena in order to stimulate a broader and more interdisciplinary reflection on the interactions between nanostructured materials and human skin. Firstly, we will define what a nanomaterial is and why it is important to study its interaction with the skin compared to traditional macroscopic materials. Subsequently, in the following sections, we will address, separately, how nanocarriers interact with the skin in the case of drug delivery and how micro- and nanoplastics have become among the most important emerging pollutants. We will then focus on which physicochemical parameters (size, shape, and surface functionalization) influence the absorption of these nanomaterials through the skin. Finally, we will provide some considerations regarding the future evolution of this phenomenon.

## 2. Introduction

**Figure 1:**
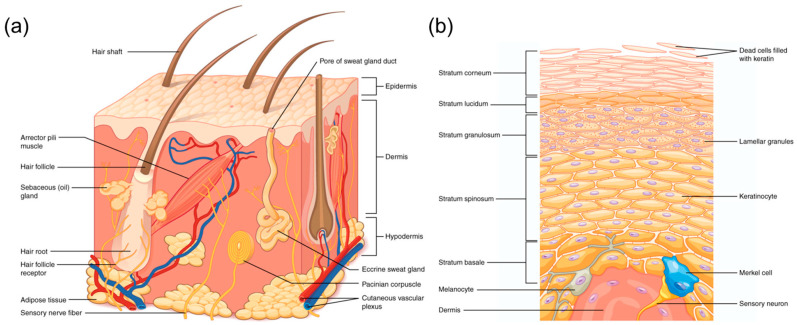
(**a**) The skin is composed of two main layers: the epidermis, made of closely packed epithelial cells, and the dermis, made of dense, irregular connective tissue that houses blood vessels, hair follicles, sweat glands, and other structures. Beneath the dermis lies the hypodermis, which is composed mainly of loose connective and fatty tissues. (**b**) The epidermis of thick skin has five layers: Stratum Basale, Stratum Spinosum, Stratum Granulosum, Stratum Lucidum, and Stratum Corneum. (Reported with permission from ref. [2]. Copyright Lumen Learning).

Skin represents the largest human organ and the boundary of the human body exposed to the external world. Skin behaves as a natural barrier that strongly limits the penetration of external agents to avoid invasion by pathogens and potentially dangerous external bodies [1]. This limited permeability is given by its multilayered structure, schematized in Figure 1 [2]. Skin is composed of three different layers: the epidermis, dermis, and hypodermis. The external layer, the epidermis, is itself a multi-layered structure composed of the Stratum Corneum, Stratum Lucidum, Stratum Granulosus, Stratum Spinosum, and Stratum Basale. The most external layer, the Stratum Corneum, represents the principal barrier against the penetration of external agents, also showing the withstanding of mechanical force. Communication between the different layers is complex and can in part alter the permeability of the skin. The skin barrier is reinforced by various factors, including the acidic mantle (rich in lactate and free fatty acids) that inhibits bacterial growth and maintains skin pH [3]. Ceramides, the primary lipids of the Stratum Corneum, form a hydrophobic barrier, preventing water loss and protecting against environmental insults. Additionally, ceramides safeguard mitochondria, ensuring adequate energy supply for epidermal functions [4]. Furthermore, immunological factors significantly influence skin health and function. Hormonal or immune imbalances can cause conditions like psoriasis and inflammation, disrupting skin structure and increasing permeability [5]. Because of its structure, the permeability of skin strongly depends on the sizes of the potentially penetrating objects. Their shape and surface properties also play a role. “Large” objects can hardly pass through skin unless they are able to create a breakage, like in the case of needles, which can be used to inject substances with therapeutical or esthetical effects through the skin. On the other hand, small molecules can pass the derma with an efficiency that strongly depends on their chemical and physical properties. Ideally, an intracutaneous delivery system should effectively solubilize a drug and facilitate its permeation through the Stratum Corneum. Concentration is crucial as excess can lead to drug precipitation and reduced bioavailability. Additionally, the partition coefficient between the carrier and skin influences drug release, with excessive solubility in the carrier hindering transport to the Stratum Corneum. The permeability of skin to nanosized and microsized particles, which are much larger than molecules but smaller than macroscopic objects, is becoming a relevant concern.

Quite surprisingly, the issue of the penetration of micro- and nanoparticles through the skin presents a dual valence. On one side, (i) nanoparticles can be used as vectors for the delivery of active molecules through the skin with a therapeutical function; on the other side (ii) nano- and microplastic particles represent an emerging example of environmental pollution [6]. Additionally, passing through the skin, pollutant particles can transport and deliver toxic molecules, increasing their actual hazards for human beings. Despite the very different scenarios, understanding the mechanisms of penetration of nano- and microparticles is of primary importance both for improving therapeutical or diagnostic applications and for mitigating the potential toxicity of pollutant plastic particles. In this review paper, we critically discuss the most recent publications about micro- and nanoparticles passing through the skin, underlining how this process can be seen either as an advantageous drug delivery method or as an unwanted path for accumulating pollutants in the human body. In the first place, we will elucidate the difference between macroscopic, microscopic, and nanoscopic materials, highlighting how the size of a material can significantly influence its properties. We will then focus on how these considerations affect the study of nanoparticles and nanoplastics. Subsequently, we will delve into the potential harmfulness of such particles and, following this, their potential benefits. We will continue by discussing how nanoplastics are becoming some of the most widespread pollutants on the Earth and how this could prove to be a critical problem in the coming years. Finally, we will examine two different nanomaterials: PLA/PLGA nanoparticles as drug delivery systems and polystyrene nanoplastics. For each of these, we will attempt to understand how certain characteristics (size, shape, and surface chemical properties) can influence transdermal penetration mechanisms.

## 3. Microplastics, Nanoplastics, and Nanoparticles

In this first section, we will explore the characteristics and origins of micro- and nanomaterials. A nanomaterial is defined as a material in which at least one dimension is smaller than 100 nm, although, in some cases, this term is extended to include all materials with at least one dimension smaller than 1 μm [7]. On the other hand, a micromaterial refers to sizes between 1 mm and 100 nm [8]. We will focus on microplastics and nanoplastics, responsible for one of the biggest environmental issues because of their high polluting capacity, and we will also address the topic of engineered nanomaterials, particularly in the biomedical field. Since Richard Feynman first introduced the concept of “nanotechnology” into contemporary science in his famous speech “There’s plenty of room at the bottom” in 1959, research in the field of nanoscience has undergone rapid evolution [9]. Indeed, scientists soon discovered that in the so-called “nanoscale world”, there was a new universe to be explored [10]. In this regard, when considering the physics and interactions of materials at the nanoscale, the rules of classical physics lose their validity and the behavior of materials is dictated by quantum physics. For example, moving from the macro- to the nano-world, the effect of gravity always contributes less to a system while weak interactions between molecules become some of the main driving forces [11]. This means that a material can have different properties depending on whether it is considered in its macroscopic or nanoscopic form. This finding was revolutionary for several reasons: for example, it was realized that potentially any currently known material could hide different, useful, and interesting properties if miniaturized in nanoscale form [12]. Consequently, it multiplied enormously, and new paths were available for the production of new materials with designed properties. Finally, it has been realized that nanotechnology can have a significant impact on the field of molecular biology since the physiology of cells and tissues is primarily regulated by biomolecular machines. Consequently, nanotechnology investigation techniques can greatly help us explore the mechanisms and functions that affect the life and health of living beings, allowing us to better understand how the machinery of life works and how to treat it when necessary [13].

**Figure 2:**
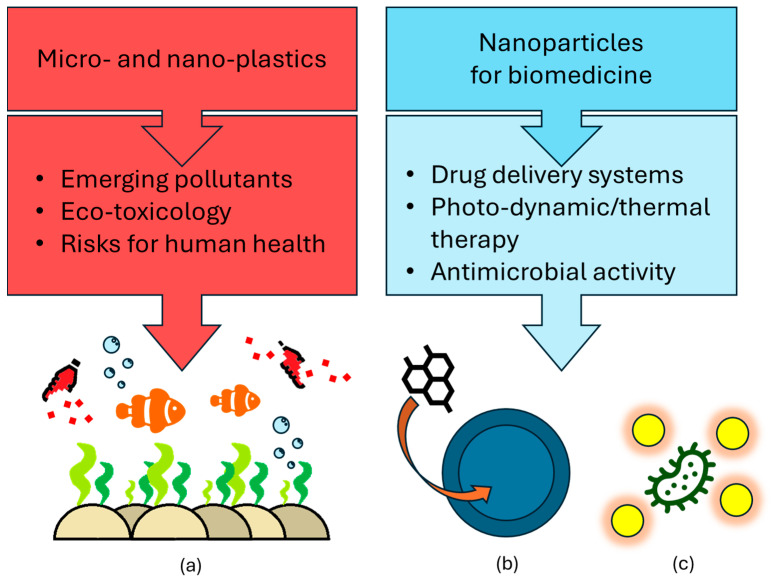
Schematic representation of (**a**) micro- and nanoplastics and their roles as emerging pollutants and in nanoparticle biomedicine and their application as (**b**) drug delivery systems or (**c**) antimicrobial agents.

Micro- and nanomaterials can form both spontaneously, such as from the breakdown of macroscopic materials, and artificially, as in the case of the synthesis of engineered nanomaterials in a chemical laboratory [14,15]. For example, micro- and nano-pollutants are ubiquitous in the environment, originating from a diverse range of anthropogenic activities and natural processes [16]. For example, microplastics and nanoplastics pose a significant threat to marine ecosystems. The origins of these pollutants are multiple and due to poor waste management: this practice results in the substantial input of plastic waste into the global marine ecosystem [17]. Micro- and nanoplastics primarily originate from the breakdown of larger plastic garbage items due to factors such as sunlight, wave action, and interactions with marine environments (Figure 2a). In addition, a significant portion of micro- and nanoplastics entering aquifers originate from products designed for direct human use. Synthetic fibers shed from clothing during washing and microplastic particles from personal care products are notable examples [18]. The earliest recorded instances of the phenomenon can be traced back to the 1960s, characterized by the discovery of plastic fragments within marine fauna. The term “microplastic” was introduced into the scientific literature in 2004, and by 2008, the issue had garnered sufficient attention to be included in policy discussions [19]. This heightened awareness has culminated in bans on certain microplastics imposed by various regulatory agencies [20]. In particular, since the last decade, several nations have proposed or implemented bans on the use of microplastics in everyday products such as cosmetics. For example, since 2015, the United States has prohibited the use of microplastics, known as microbeads, in ‘rinse-off’ products. In subsequent years, countries like South Korea, Taiwan, Canada, France, New Zealand, Sweden, and Italy have adopted similar measures [20]. Although nanomaterials present potential risks to both human health and the environment, their unique properties have led to groundbreaking advancements in various fields, especially medicine. The term ‘nanomedicine’ highlights the significant potential of nanotechnology in treating diseases. Nanomaterials have played a crucial role in transforming the healthcare field [21]. They have facilitated the development of innovative drug delivery systems, advanced cancer therapies such as photodynamic and photothermal treatments, and novel antimicrobial agents (Figure 2b,c) [22,23,24,25]. Indeed, transdermal drug delivery has evolved over time. First-generation systems, such as creams and patches, were primarily used for delivering small, lipid-soluble molecules. Second-generation systems introduced techniques like iontophoresis and ultrasound to enhance skin permeability. The third generation focused on overcoming the barrier of the Stratum Corneum using microneedles. The latest advancements involve the use of micro- and nanoparticles as drug carriers [26]. The next sections will provide a comprehensive overview of the toxicological profiles of nanomaterials and explore their promising applications in the biomedical domain, including drug delivery.

## 4. Toxicity of Microparticles and Nanoparticles

**Figure 3:**
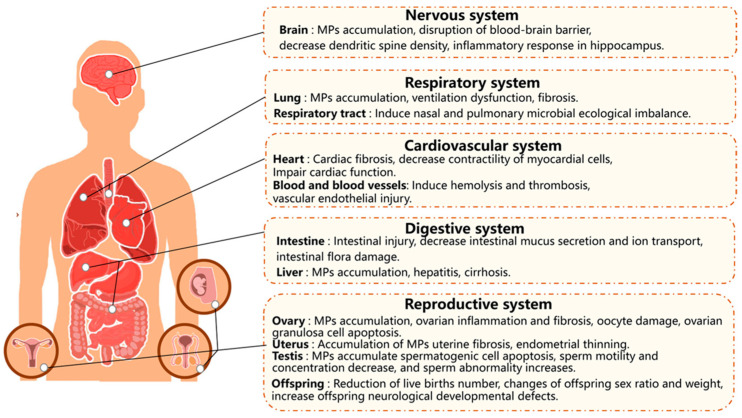
Microplastics: toxicity in the human body. (Adapted with permission from ref. [34]. Copyright 2023, Elsevier).

The advent of research in micro- and nanomaterials has led parallelly to the exploitation of their potential in many fields and to the investigation of their potential toxicity. One of the first papers that highlighted the urgency of a new toxicological science related to nanomaterials was “Toxic potential of materials at the nanolevel”, published in *Science* by Nel et al. in 2006 [27]. Here, the authors stressed that the size and the surface properties of nanoparticles are the main factors responsible for their potential toxicity. On the other hand, the size and surface represent the main peculiarities that distinguish a nanosized material from a bulk material, which is why nanosystems have been employed in so many applications. For this reason, investigation for the applicability and toxicity of nanosized materials needs to proceed in parallel. Along with nanoparticles, also, microparticle toxicity has started to be an object of study, mainly about the exploration of microplastics, the main concern of plastic material degradation in the environment [28,29]. The presence of nano- and microparticles in an organism can damage many human tissues, cells, and apparatuses. These materials have been demonstrated to bring energy homeostasis, intestinal disturbance, immunotoxicity, neurotoxicity, genotoxicity, cardiovascular toxicity, and cytotoxicity (Figure 3) [30,31,32,33].

These effects are mainly due to the ingestion and inhalation of micro- and nano-pollutants, and skin contact is usually considered a minor internalization mechanism [34]. However, penetration through the skin, mostly limited to nanoparticles, has been confirmed, and thus, this pathway should not be ignored [29]. In general, the skin penetration of micro- and nanomaterials is being more explored for drug delivery purposes than for toxicity issues. However, in the optics of having a complete perspective on the characteristics of the produced micro- and nanomaterials, for example in cosmetics, it would be necessary to treat these systems in parallel: both as drug delivery agents and as pollutants.

## 5. Nanoparticles and Microparticles for Drug Delivery

**Figure 4:**
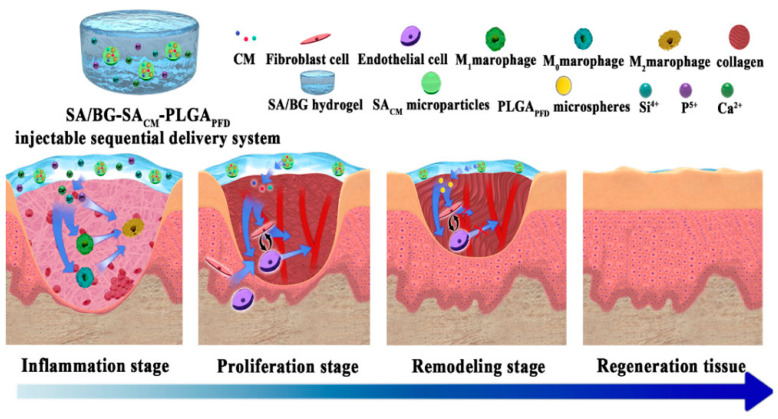
SA/BG-SA_CM_-PLGA_PFD_ delivery system action during the wound healing stages. (Reported with permission from ref. [50]. Copyright 2020, American Chemical Society).

As described in the previous sections, the penetration and circulation of micro- and nano-systems in the body are investigated for two purposes: as toxicity agents and as vehicles in nanomedicine. In this section, we will discuss recent advances in using micro- and nanoparticles for drug delivery that exploit skin penetration. Oral delivery and intravenous injections are the most common delivery methods, which, however, present some drawbacks. Drugs orally delivered are subjected to possible degradation due to enzymatic reactions and acid environment in the stomach [35] while the intravenous injection of drugs is an invasive method, with higher costs due to waste disposal [36]. In this context, skin penetration has become a valid alternative for being less invasive, for avoiding metabolism-induced drug degradation, and thanks to its large area for drug absorption [37]. Nevertheless, drug penetration through the skin is particularly challenging because of the presence of the Stratum Corneum, the epidermal barrier of 10–20 μm composed of corneocytes (derived from the assembly of dead keratinocytes) and glycolipids in the intracellular space [38,39,40]. For this reason, using nanocarriers is essential to transport drugs through the skin barrier, increasing their solubility and stability. Nanometer-sized carriers are employed to enhance permeability, and they usually are lipid-based [41,42], polymeric [43,44], and some inorganic nanoparticles [45]. Nowadays, lipid nanoparticles are widely used in drug delivery since they improve the solubility of drugs in the organism. Liposomes are the most common lipid nanoparticles, and some liposome-based systems are already used in clinical trials. An example of liposome use in a drug delivery system with transdermal penetration was given by Lee et al. [46]. Here, liposomes were used to transport indocyanine green (ICG) for the photodynamic therapy of melanoma. In particular, liposomes were coated with a cationic polymer, chitosan, to increase ICG stability and skin permeation. The liposomes obtained had a size of 150–200 nm each and the incorporation of ICG did not lead to changes. However, the addition of chitosan led to ICG-loaded liposome aggregation. Skin permeation tests, performed using the Franz-type vertical diffusion cell protocol [47], showed similar behaviors in free ICG and ICG embedded in uncoated liposomes. Moreover, in both cases, ICG was just distributed in the upper layer of the epidermis because of its hydrophilicity. The coating of the liposomes with chitosan, despite the bigger size of the system due to aggregation, permitted an increased ICG permeation, probably due to the strong adhesion of chitosan to the skin. Among lipid-based systems, ethosomes usually have enhanced skin permeation. Ethosomes are phospholipid nanoparticles with 20–45% of ethanol. Ethanol contributes to permeability and elasticity increase, which favor the interaction with the Stratum Corneum [22]. For example, Aodah et al. embedded hexatriacontane (HTC) in transethosomes (TESs) to treat microbes’ skin infections [48]. The use of a HTC-loaded TES gel, composed of lipoid, ethanol (25%), and sodium cholate, favored the antimicrobial efficacy after topical deposition on rat skin, with higher performances than the use of a conventional gel for HTC loading. Polymer-based nanosystems are also commonly used for delivering drugs through the skin. Common polymeric nanoparticles surely are the ones based on poly (lactic-co-glycolic) acid (PLGA) because of their biodegradability and biocompatibility [49]. Ma and co-workers used PLGA nanospheres in a multi-layer system for the treatment of wound healing stages [50]. They developed a hydrogel based on sodium alginate and bioglass (SA/BG) to act in the early inflammatory stage, carrying SA microparticles with a conditioned medium of cells (CM), useful for the proliferation stage. They embedded PLGA microparticles loaded with pirfenidone (PFD) inside this system to regulate the extracellular matrix synthesis and inhibit angiogenesis, accelerating the skin regeneration process (Figure 4).

**Figure 5:**
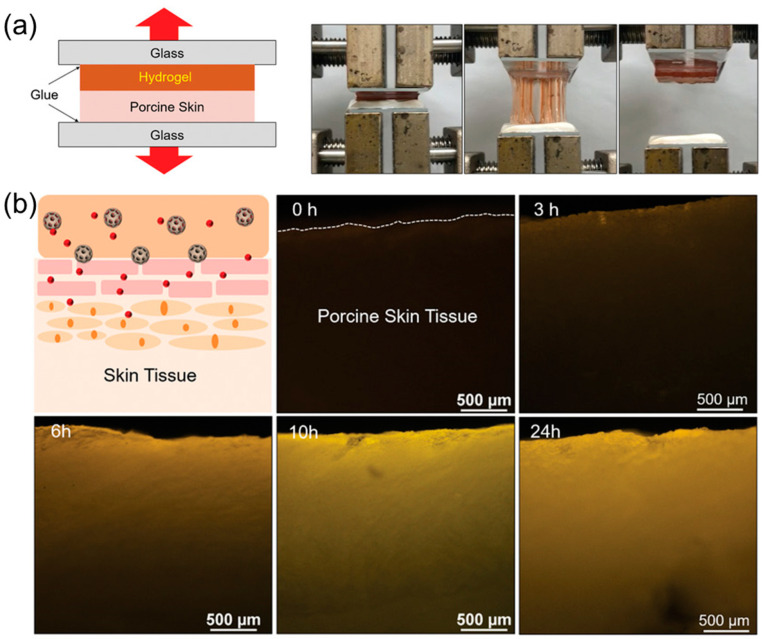
(**a**) Scheme and photos of tensile adhesion test on the PAM/PDA/XL-MSN hydrogel to the porcine skin. (**b**) Scheme of the internalization process of R6G (red dots) from PAM/PDA/XL-MSN through the porcine skin tissue and its fluorescence microscope imaging in the time range of 0–24 h. (Adapted with permission from ref. [53]. Copyright 2020, John Wiley and Sons).

Here, PLGA microparticles need to act at the last stage of the wound healing process: this is possible because of the dense structures of the polymeric microparticles, which delay their degradation and the release of the drug. As shown in the papers discussed above, hydrogels are widely used in transdermal drug delivery. This is due to their high biocompatibility, loading capacity, ease of application, and their biophysical similarity to natural tissues [51,52]. Another example of a polymeric hydrogel used for drug delivery was described in the paper of Jung and co-workers [53]. They designed a polyacrylamide/polydopamine (PAM/PDA) hydrogel embedded with extra-large-pore mesoporous silica nanoparticles (XL-MSNs) for transdermal drug delivery. The combination of PMA, largely used, and PDA was to increase the adhesiveness, exploiting the adhesive properties of the catechol groups of PDA, which interact with the imidazole moieties present on the surface of the skin. However, this composite hydrogel compromises the mechanical properties because of a loss of cohesion due to the presence of PDA. For this reason, XL-MSNs were added to the hydrogel structure: they not only incorporate the drug but also improve the cohesive forces in the hydrogel thanks to further hydrogen bonds. In this way, the adhesiveness of the final system was significantly improved and the drug release was favored. Tensile-adhesion tests, performed with porcine skin tissue, showed an adhesive energy that was three-fold higher in the PAM/PMA/XL-MSN hydrogel compared to the PAM/PDA one, confirming the improvement of cohesion within the hydrogel (Figure 5a).

Then, transdermal drug delivery was confirmed with the incorporation of a dye (Rhodamine 6G) in XL-MSN (Figure 5b). The fluorescence of the dye was monitored for 24 h, reporting good penetration in the tissue (Figure 5b). As shown in the previous examples, drug delivery through skin penetration depends on several factors. The overcoming of the Stratum Corneum is a central point for obtaining an efficient drug release. In addition, also, the lifetime of the encapsulated drug and the adhesion on the skin surface are significant parameters that deeply influence the action of drugs for topical skin treatments. Beyond these aspects, there always is the necessity to use biocompatible and biodegradable systems to avoid or at least minimize the toxicity of these materials.

## 6. Nanoparticles and Microparticles as Pollutants

**Figure 6:**
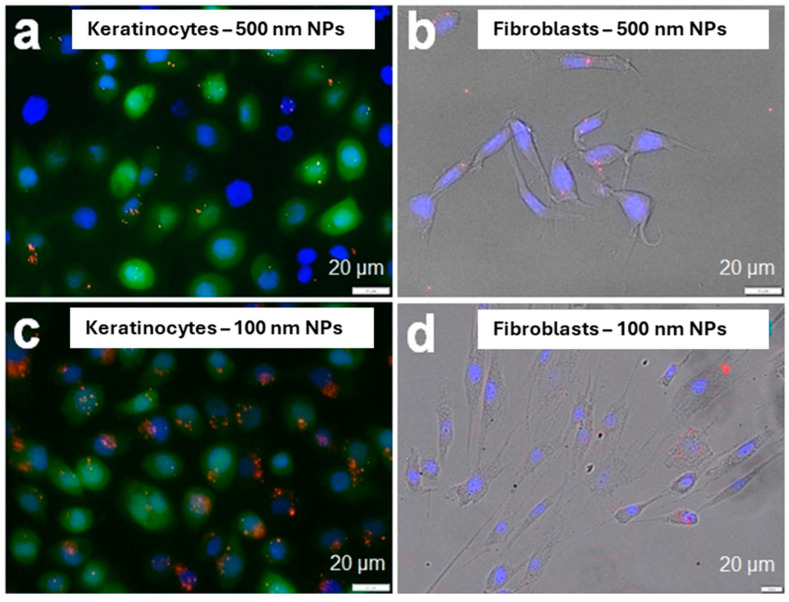
Accumulation of 500 nm nanoparticles in keratinocytes (**a**) and fibroblasts (**b**); accumulation of 100 nm nanoparticles in keratinocytes (**c**) and fibroblasts (**d**). (Adapted with permission from ref. [64]. Copyright 2024, Springer Nature).

In the previous section, we described the use of nano- and microparticles as drug delivery agents. Here, the design of these materials is focused on achieving efficient skin penetration, using, when possible, biocompatible and biodegradable materials. However, due to their toxicity, nano- and microparticles are also studied as pollutants. In this case, the aim of the investigation is the detection of these materials. The techniques used for the nano- and microparticle detection can go from gas chromatography/mass spectrometry (GC/MS) and differential scanning calorimetry (DSC) to fluorescence and Raman microscopy, and electron microscopy, the microscopy techniques usually more sensitive to smaller-sized analytes [54,55]. Among these materials, most research is focused on micro- and nanoplastics, which constitute most of the micro- and nanosized pollutants present in the environment. The detection of micro- and nanoplastics is mainly investigated in (1) water (river or lake drinking water, and seawater) [56,57] and the atmosphere [18,58] and in (2) human biological samples (blood, urine, lung tissue, breast milk, etc.) [30,59]. Research on the detection of micro- and nanoplastics through skin penetration is not frequent. Dermal interaction with micro- and nanoplastics can occur through contaminated water or cosmetic products [60], and particles bigger than 100 nm usually do not cross the Stratum Corneum [61]. This limits the penetration issues to nanoplastics and makes skin adsorption less relevant than ingestion and inhalation, considered the main pathways for the internalization of these pollutants. However, despite limited long-term data, there is evidence suggesting that the dermal uptake of microplastics and nanoplastics could induce cutaneous alterations, inflammation, and disruptions to the skin’s physiological homeostasis [62]. Furthermore, systemic exposure to nanoparticles has been linked to long-term health risks including metabolic disturbances, adverse immune responses, neurotoxicity, reproductive disorders, and developmental abnormalities [63]. A recent interesting point of view was described in the paper of Martin et al. [64]. They evaluated the skin penetration of nanoparticles simulating a situation in which the skin barrier was compromised. As mentioned, the main obstacle to the internalization of nanomaterials through the skin is given by the presence of the Stratum Corneum. Several factors such as skin inflammation, injury or allergy [65,66], age [67], and the use of aggressive detergents can cause the removal of the Stratum Corneum, making the skin more sensitive and permeable to micro- and nano-agents. In their paper, Martin and co-workers evaluated the penetration of fluorescein-stained polystyrene nanoparticles, as models of nanoplastic particles, of 500 nm and 100 nm. They first tested the uptake of these systems in keratinocytes and fibroblasts, present in the epidermal and dermal layers, respectively (Figure 6). In particular, both nanoparticles were found in keratinocytes and fibroblasts after 16 h of incubation, but the presence of 500 nm nanoparticles was more limited than that of 100 nm nanoparticles. Both kinds of nanoparticles accumulated to form clusters in both keratinocytes and fibroblasts.

Then, they compared the penetration of the particles in the presence and absence of the Stratum Corneum using ex vivo skin models. As expected, this test showed a significantly higher penetration of nanoparticles in the skin model not protected by the Stratum Corneum. Finally, they monitored the permeation of the nanoparticles from the epidermis to the fibroblasts in the dermis using a 3D skin model. They noticed a penetration of the nanoparticles also in the fibroblasts, i.e., in a more internal layer of the skin. These results confirmed the enhanced penetration of nanoparticles through the skin when the Stratum Corneum is removed. However, skin penetration is surely highly dependent on the composition of the nanomaterial and its surface properties. In this work, the study was limited to polystyrene, modified with fluorescein that altered its surface properties. In general, considering the importance of this environmental topic, and the large use of cosmetics that could act as vehicles for micro- and nanosized pollutants, a more systematic investigation comparing different materials and surface properties would improve knowledge of this field and the more appropriate design of new cosmetic systems.

## 7. Effects of Size, Shape, and Surface on Penetration Through the Skin

In the following section, we will focus on understanding and elucidating the physicochemical characteristics that influence the internalization of micro- and nanoparticles through the skin. The integumentary system, a complex set of organs designed to protect the body from external threats, poses a significant challenge to researchers studying the penetration of chemicals. Despite its role as a barrier, various chemicals can be absorbed through the skin. Understanding the mechanisms underlying this process is complicated by the skin’s intricate structure and composition, as discussed in the ‘Introduction’. While complex, four primary pathways for nanomaterial dermal absorption have been elucidated. It is important to note that multiple pathways may be utilized for a given nanomaterial, with the specific route often influenced by its physicochemical properties. The transappendageal route, involving hair follicles and sweat glands, represents two major pathways for dermal penetration. These appendages provide relatively wide passageways through the Stratum Corneum, facilitating the transport of molecules and nanoparticles. Furthermore, they can be accessing routes to the lymphatic system and bloodstream. Nevertheless, their limited surface density means that most substances primarily interact with the Stratum Corneum’s external surface. The alternative pathways are collectively termed the “transepidermal route”, encompassing penetration through phospholipid membranes and the Stratum Corneum. The primary pathway involves the Stratum Corneum, which consists of corneocytes containing a high concentration of hydrated keratin. This route is favored by hydrophilic substances; however, the lipophilic nature of cellular membranes hinders this passage, limiting it to a few chemical species. The final pathway, the “intercellular pathway”, involves diffusion through the lipid-rich matrix between skin cells. While lipophilic molecules preferentially utilize this route, the tortuous nature of the intercellular spaces and variations in lipid composition can impede diffusion [68]. Despite a lack of comprehensive studies on the mechanisms of skin absorption of micro- and nanoplastics, it is undeniable that these particles are ubiquitous in the environment. Their prevalence in air, water, soil, and even food makes the hypothesis of their skin absorption plausible [69]. The ubiquitous presence of these particles in the environment necessitates a more in-depth analysis of the potential risks to human health. Specifically, we will dedicate our review to two important materials that are implied in two relevant research fields: biomedicine and eco-toxicology. We are referring to polylactic acid/poly(lactic-co-glycolic) acid (PLA/PLGA) for the former case while polystyrene (PS) will be examined for the latter. It is well known that merely knowing the chemical compositions of two materials does not allow us to unequivocally predict how they will interact [70]. Other factors, such as shape, size, and surface characteristics, significantly influence this process [71,72]. We will delve deeper into these characteristics for the specific materials under investigation. This consideration is particularly crucial when dealing with nanomaterials, as it has been well established that the size of a material can dramatically affect its properties. Consider gold, for instance: while macroscopic gold exhibits a characteristic yellow metallic color, gold nanoparticles can appear from red to blue depending on their sizes, shapes, and surface functionalization [73,74]. From the earliest studies of nanomaterials in life sciences, it has become evident that their interaction with biological tissues differs significantly from that of traditional materials [75]. Nanomaterials approach the sizes and shapes of cells and the molecular machines within them that regulate vital and physiological functions [76]. This similarity renders biological tissues more reactive to nanomaterials compared to macroscopic materials [77]. Despite this, in recent years, nanomedicine has led to astonishing discoveries regarding human health, and it is no surprise that nanotoxicology is currently one of the hottest topics in international research [78]. Given these findings, it is not unexpected that regulatory frameworks have been established to control the incorporation of nanomaterials into commercial products including goods like cosmetics [79]. Therefore, it is evident that understanding the intrinsic properties of skin and the nanomaterials with which it interacts is crucial for elucidating their interaction mechanisms.

### 7.1. PLA and PLGA

**Figure 7:**
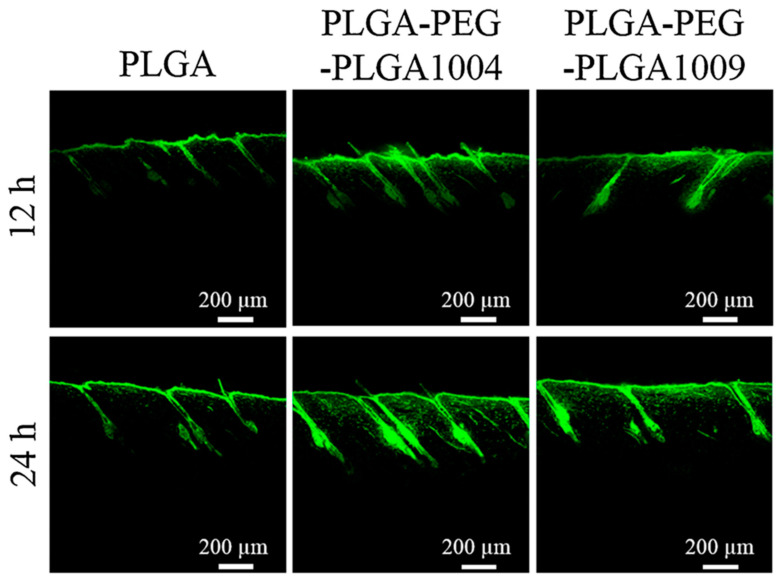
Confocal laser microscope images of cross-sections of the rat skin of coumarin-6-loaded PLGA, PLGA-PEG-PLGA1004 (Mw: 4000/1000/4000, monomer composition of dl-lactic acid/glycolic acid/ethylene oxide = 62/22/16), and PLGA-PEG-PLGA1009 (Mw: 3500/4000/3500, monomer composition of dl-lactic acid/glycolic acid/ethylene oxide = 48/13/39) nanoparticles at 12 and 24 h from the initiation of the ex vivo skin permeability tests. (Reported with permission from ref. [91]. Copyright 2020, Elsevier).

Polylactic acid (PLA) and poly(lactic-co-glycolic) acid (PLGA) are prominent bioplastics due to their low environmental impact and biocompatibility [80,81]. PLA is derived from lactic acid esterification while PLGA is a copolymer of lactic and glycolic acids [82]. Their chemical precursors, produced via biotechnological methods like bacterial fermentation, can be efficiently metabolized by microorganisms [83]. PLA and PLGA’s biocompatibility and resorption make them suitable for biomedical applications. They are used in tissue engineering, such as in creating artificial tendons and bone regeneration scaffolds [84,85,86]. Additionally, their properties enable them to serve as nanocarriers for drug and bioactive compound delivery, including transdermal applications [87]. As we will see in the next lines, the penetration and efficacy of these nanoparticles within the skin are influenced by factors such as the particle size and surface properties. Also, their internalization varies depending on the application site (e.g., epidermis, hair follicle). Regarding the effect of nanoparticle size, several studies can be cited. For instance, Lin et al. focused on a size-dependent study of PLGA nanoparticles loaded with two anti-psoriasis drugs, 3,3′-dioctadecyloxacarbocyanine perchlorate and 1,1′-dioctadecyl-3,3,3′,3′-tetramethylindocarbocyanine perchlorate [88]. The study employed two populations of nanoparticles: a smaller group with an average diameter of approximately 50 nm and a larger group with a diameter of around 226 nm. Interestingly, skin dendritic cells exhibited a preferential uptake for the larger nanoparticles compared to the smaller ones. Moreover, the intracellular fates of the nanoparticles differed based on their sizes. Smaller nanoparticles were distributed simultaneously to the skin-draining lymph nodes and the spleen while larger nanoparticles were initially internalized by the skin-draining lymph nodes before being subsequently transported to the spleen. Nevertheless, it is imperative to acknowledge the skin’s intricate anatomical structure. Investigations have demonstrated a correlation between the dimensions of nanocarriers and their cutaneous penetration capacities, contingent upon the specific region of the integumentary system under scrutiny. A noteworthy investigation by Kshirsagar et al. explored the cutaneous penetration of tazarotene-loaded PLGA nanoparticles [89]. The study demonstrated a differential uptake of these nanocarriers across distinct epidermal regions. Notably, hair follicles facilitated the internalization of nanoparticles in a size-discriminate manner, with smaller nanoparticles (with a diameter of around 290 nm) exhibiting enhanced uptake compared to their larger counterparts (with a diameter of around 500 nm). Conversely, the permeation of nanoparticles through the Stratum Corneum was found to be independent of their size. A comparable trend was documented by Takeuchi et al. [90]. The investigators evaluated two disparate populations of PLGA nanoparticles, one with a mean diameter of 50 nm and the other of 100 nm. These nanocarriers were subsequently loaded with compounds such as indomethacin and coumarin-6. The study revealed a preferential uptake of smaller nanoparticles through hair follicles relative to their larger counterparts. Consistent with the aforementioned study, internalization through the Stratum Corneum was limited, although enhanced nanoparticle penetration was observed upon the application of iontophoresis. In addition, the surface functionalization of PLGA nanoparticles emerges as a pivotal factor influencing their cutaneous penetration. In alignment with this, Takeuchi et al. documented the enhanced Stratum Corneum permeation of PLGA nanoparticles following conjugation with hydrophilic PEG polymer chains [91]. This augmentation was attributed to PEG’s capacity to inhibit interactions between the nanoparticles and cutaneous proteins, thereby mitigating their propensity to adhere to the nanoparticle surface and to impede deep epidermal penetration (Figure 7).

To support this, we can mention that Özcan et al. reported the differential cutaneous penetration of hydrophilic and lipophilic nanoparticles [92]. The investigators compared the permeation of two distinct nanocarriers, considering the transport through hair follicles and intercellular lipid channels within the skin. The study encompassed PLGA and lecithin/chitosan (LC) nanoparticles. The findings revealed a preferential internalization of PLGA nanoparticles within the dermis, attributable to their enhanced capacity for follicular uptake. Conversely, the hydrophobic nature of lecithin nanoparticles facilitated their accumulation in the epidermal stratum, with limited dermal penetration. In corroboration of these findings, Laredj-Bourezg et al. investigated poly(lactide)-block-poly(ethylene glycol) copolymer (PLA-b-PEG) and poly(caprolactone)-block-poly(ethylene glycol) copolymer (PCL-b-PEG) nanoparticles as vectors for the cutaneous delivery of hydrophobic molecules including retinol and Nile red [93]. The study revealed the superior transport efficiency of PLA-b-PEG nanoparticles compared to their PCL-b-PEG counterparts. While the investigation primarily centered on the influence of guest molecule solubility within the nanocarriers on cutaneous penetration, it is noteworthy that the hydrophilic character of PLA, conferred by its polar functional groups, may also contribute to its enhanced transport efficacy through hair follicles.

### 7.2. Polystyrene

**Figure 8:**
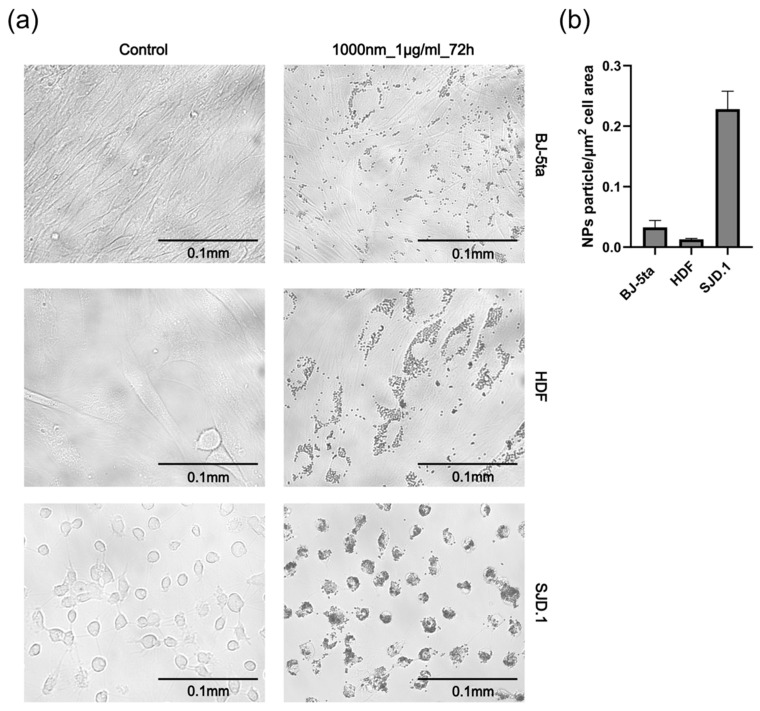
(**a**) Microscope images and morphological observation of fibroblast cell lines with and without PS nanoparticles (size = 1000 nm at 1 μg/mL, 72 h after application). (**b**) A graphical representation of the number of PS nanoparticles taken up by cells. (Reported with permission from ref. [104]. Copyright 2024, Elsevier).

Polystyrene (PS), a versatile synthetic polymer, is widely utilized due to its exceptional properties [94]. Its amorphous structure imparts transparency, rigidity, and excellent electrical insulation [95]. Additionally, its hydrophobic nature renders it resistant to moisture. These attributes make polystyrene an ideal material for packaging and structural support [96]. Expanded polystyrene, derived from the packing of polystyrene beads, exhibits low density, superior thermal insulation, and effective shock absorption, making it a preferred choice in construction and transportation applications [97]. The global polystyrene market size was estimated at USD 34.55 billion in 2023. It is projected to reach USD 60.10 billion by 2034, expanding at a CAGR of 5.16% [98]. Such consumption has also made it one of the most environmentally impactful plastics globally. Indeed, polystyrene fragments in the form of micro- and nanoplastics have been found in both freshwater and marine environments [99,100]. For example, small PS microplastics (1–100 μm) and nanoplastics (<1 μm) were detected in the Bohai Sea, with mass concentrations ranging from <0.015 to 0.41 μg/L [101]. Additionally, the bioaccumulation of microplastics in biological tissues has facilitated their transfer through the food chain [102]. The bioaccumulation of micro- and nanoplastics is a relatively new concern, and the underlying mechanisms and long-term consequences remain unclear. The specific pathway of polystyrene micro- and nanoparticle internalization through the skin is a particular area of interest. The following studies explored how the size, shape, and surface properties of polystyrene micro- and nanomaterials influence their uptake by cells and tissues of the integument. About the size effect, Schmidt et al. isolated and cultured dermal fibroblasts and epidermal keratinocytes from murine rats [103]. These cultured cells were subsequently exposed to polystyrene microplastics of different sizes: 0.2 μm, 1.0 μm, 2.0 μm, 6.0 μm, and a heterogeneous mixture ranging from 1 to 5 μm. The research findings indicated that the smaller microplastic fragments, particularly those measuring 1.0 and 2.0 μm, exhibited the highest level of accumulation within the cells. However, cell viability tests did not show a strict correlation between particle size and toxicity. In accordance with such results, Peng et al. conducted a similar experiment, testing polystyrene nanoparticles of varying sizes (0.05, 0.5, and 1.0 µm) on dermal fibroblasts from different organisms (zebrafish SJD.1, human male newborn BJ-5ta, and female adult HDF) [104]. The study revealed that the effects on cell proliferation are dependent on the sizes and concentrations of the micro- and nanoparticles to which the cells are exposed. It was observed that the inhibitory effect of nanoparticles on proliferation was more pronounced in human skin fibroblasts compared to fish fibroblasts, where only nanoparticles of 1.0 µm had a significant impact (Figure 8).

Notably, as the sizes and concentrations of polystyrene micro- and nanoparticles increased, the inhibitory effect on fibroblast proliferation became more pronounced. Furthermore, the results with skin fibroblasts indicated that larger nanoparticles at higher concentrations caused more significant inhibitions of cell migration. The distinct outcomes induced by micro- and nanoparticles could be attributed to their different modes of interaction with skin cells. In this regard, Akhatova et al. assessed the adhesion capacity and penetration of polystyrene micro- and nanoparticles into human skin fibroblasts to elucidate the mechanisms underlying their cytotoxicity [105]. Through dark-field hyperspectral microscopy and atomic force microscopy, the researchers found that particles with a size of 0.5 μm or less were internalized by fibroblasts. Conversely, 1.0 μm microparticles were only localized on the cell surface. This difference could also explain the varying adverse effects reported in previous studies. As is well known, in vitro and single-cell studies can provide valuable insights into the biological mechanisms underlying various bodily processes. However, these approaches are insufficient. To fully elucidate the physiological interactions between micro- and nanoplastics, more complex models that closely mimic real biological tissues are required.

**Figure 9:**
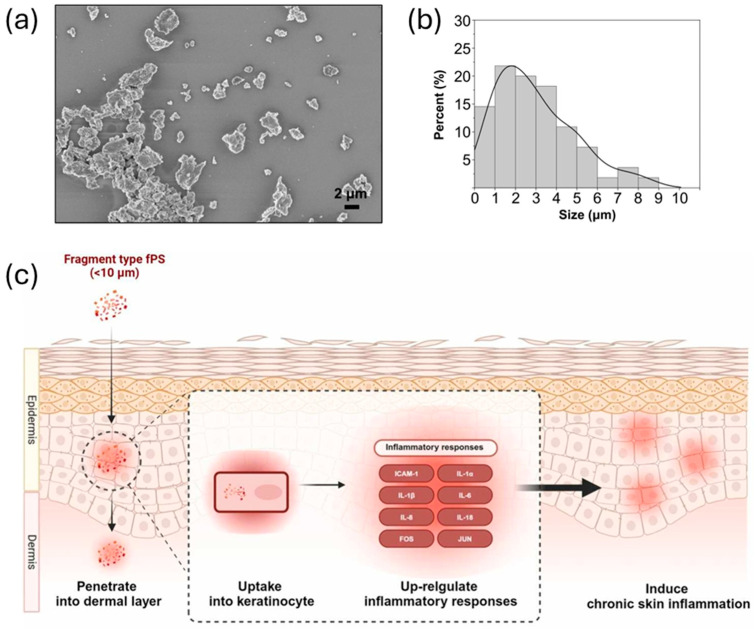
(**a**) Morphological characterization of the polystyrene fragments using field emission-scanning electron microscopy. (**b**) Size distribution of polystyrene fragments. (**c**) Schematic representation of the internalization path of polystyrene fragments in mammalian skin. (Adapted with permission from ref. [106]. Copyright 2024, Elsevier).

In this regard, Song et al. investigated the internalization of polystyrene particles using a 3D human skin model, mouse dorsal skin, and human abdominal skin [106]. The 3D model demonstrated the penetration of micro- and nanofragments with sizes of 2 μm or less (Figure 9a,b). Animal skins, on the other hand, exhibited greater selectivity, allowing the penetration of particles smaller than 1 μm. Micro- and nanoplastics were absorbed by both the epidermis and dermis but were not observed in the hypodermis (Figure 9c). Additionally, a deeper penetration was observed when a higher concentration of particles was applied to the skin. Interestingly, human skin appeared to be more permeable than murine skin. The authors hypothesized that this difference might be attributed to larger human hair follicles, which could facilitate the uptake of larger particles in greater quantities.

Previous studies have demonstrated that not only the size but also the morphology of a micro- or nanomaterial can significantly influence both its intrinsic properties and its ability to interact differently with biological cells and tissues. Regarding polystyrene microplastics, Choi et al. investigated this topic, focusing on larger microplastics (5–200 μm) compared to those examined in previous studies [107]. They specifically explored the role of microplastic roughness in influencing cytotoxicity in skin fibroblast cells. Their findings indicated that rougher microplastics tend to cause greater physical damage to cell walls, leading to hemolysis and LDH release into the cytosol. Consequently, they attested that both physical and chemical damages contribute to the harmful effects of such microplastics. Not least importantly, it is worth asking what the effect of the chemical composition of such materials is on the penetration mechanism of micro- and nanoplastics through the skin. In this regard, we can cite the research work of Cheng et al. [108]. In this study, the authors simulated the human Stratum Corneum through a Langmuir monolayer composed of ceramides, cholesterol, and fatty acids and investigated the permeability of simple polystyrene nanoparticles (PS NPs), as well as those functionalized with carboxylic (PS-COOH NPs) or amino groups (PS-NH_2_ NPs). The study showed that larger particles and at higher concentrations have a greater destabilizing effect on the lipid layer and, moreover, that PS NPs functionalized with functional groups interact better with such membranes through electrostatic interactions and hydrogen bonds, reducing their stability. In particular, a significant effect was observed for nanoparticles functionalized with amino groups (PS_NH_2_ NPs). A progressive increase in cytotoxicity from PS NPs to PS-COOH NPs and, most notably, PS-NH_2_ NPs was also observed in in vivo studies using human skin fibroblasts.

The preceding studies on the cutaneous internalization of PLGA nanoparticles for drug delivery and PS micro- and nanoparticles, summarized in Table 1, offer valuable insights into the role of physical characteristics in transdermal penetration. A comparison of these studies reveals a striking similarity: both biomedical nanoparticles and micro- and nanoplastics are influenced by the same factors during skin absorption. Specifically, size, shape, and surface chemistry are key determinants in both cases. For instance, there is a higher frequency of studies reporting that smaller nanoparticles are more capable of penetrating the skin, both through the cell wall and hair follicles, while larger nanoparticles tend to remain on the external surfaces of cells, causing cytotoxicity in any case. Furthermore, the surface functionalization of particles with specific functional groups seems to enhance their penetration. While these similarities in penetration mechanisms exist, they lead to divergent conclusions. On the one hand, nanomedicine offers hope for treating diseases through innovative and potentially more effective techniques. On the other hand, the interaction with certain nanoplastics can lead to adverse health effects. This difference highlights that it is not just the internalization, but the interaction with human physiology that sets these materials apart. Therefore, it is crucial to investigate how nanomaterials interact with the natural nanostructures of our bodies, which can be viewed as molecular machines.

### 7.3. Penetration of Nanoparticles Through Hair Follicles: Effect of Nanoparticle Size

The delivery of bioactive compounds through the skin by exploiting penetration through hair follicles is a promising strategy that offers some advantages over conventional penetration through the epidermis [109,110]. This approach was already reported in some of the examples previously discussed. The mechanism of this process was analyzed in detail [111] and resulted in being strongly enhanced by nanoparticles showing a strong size and concentration dependence [112,113,114].

## 8. Challenges and Future Perspectives

The main purpose of this paper has been to demonstrate that the penetration of polymeric particles through the skin presents a double valence. This process can be advantageously exploited for the delivery of active molecules for diagnostic or therapeutic applications, but it also represents a menace to human health when it leads to the unwanted accumulation of plastic particles, especially nanoparticles, in the human body. Although the actual toxicity of microplastics and nanoplastics in humans is still widely debated, it is widely accepted that these particles contribute to the delivery of toxic molecules that can be distributed in the environment or intervene in the process of production of the plastic itself. The contradiction between toxicity and biomedical potential is intrinsically present in all polymeric nanoparticles because all the properties (mainly size, shape, and surface) that represent their main potentialities in drug delivery efficiency also enhance their potential toxicity.

Accordingly, the low permeability of the skin to nanoparticles and even more to microplastics is surely an advantage. On the other hand, this feature becomes a disadvantage when nanoparticles are used as drug delivery agents. For this reason, the design of new nanomaterials for any biomedical application should be conducted in parallel with toxicity analyses. From both points of view, it would be necessary to control skin permeability to the nano-agents and their toxicity when used as nanocarriers.

Thus, we believe that future research will be developed in these main directions.

### 8.1. Controlling the Permeability of the Skin to Micro- and Nanoparticles 

In particular, skin permeability should be enhanced for biomedical applications but minimized in the case of exposure to an environment polluted by microplastics. Some methods are presently available to enhance the permeation of nanoparticles through the skin [115,116,117]. Indeed, the scientific literature reveals a plethora of techniques that can be coupled with the topical application of nanotechnology-based products to enhance skin penetration. These include electroporation, microneedling, physical methods such as abrasion and suction, and energy-based techniques such as ultrasound, laser, radiofrequency, and magnetic fields [118]. These findings indicate an important aspect: particle internalization through the skin is not merely a function of particle properties but is also modulated by external factors influencing both the skin and the particles. Consequently, the techniques discussed above could enhance the internalization of micro- and nanoplastics, going beyond nanoparticles specifically engineered for drug delivery. This suggests that skin lacerations, ultrasound, and electromagnetic radiation may facilitate the transport of micro- and nanoplastics into the skin, and in this sense, future research could lead to interesting considerations. Additionally, the results of the reviewed studies indicate that the characteristics of microplastics, such as size, roughness, and functional groups, particularly in larger particles, can compromise the integrity of skin cell membranes. This raises the possibility that microplastics may induce a form of skin abrasion, thereby enhancing their own uptake. On the contrary, some cosmetic products have been developed to protect skin from pollution [119,120,121]. These products have demonstrated some efficiency in protecting skin against airborne nanoparticles [122]. In our opinion, the ability of these formulations to protect skin from other kinds of nanoparticles should be tested or analogous preparations for such an application should be developed. The promising approach addresses the dermal absorption of micro- and nanoplastics involving the utilization of ‘anti-pollution’ cosmetics. Such products, already available on the market, are specifically designed to either prevent or lessen the detrimental effects resulting from exposure to environmental pollutants. These cosmetics employ a variety of mechanisms including the following: (i) inhibiting the adherence of pollutants to the skin’s surface; (ii) creating a protective barrier that interposes between the skin and pollutants, thereby preventing direct contact; (iii) enhancing the overall health of the skin to restore its natural barrier function; and (iv) incorporating active ingredients (e.g., antioxidants, anti-inflammatories) to mitigate the stress responses induced by the penetration of pollutants into the skin [123,124].

### 8.2. Controlling the Toxicity of Nano- and Microsized Particles for Drug Delivery 

The compositions, sizes, and shapes of nano- and microparticles dispersed in the environment can be hardly controlled. Exposure to mechanical forces, different chemical actions, or radiations (in particular ultraviolet) alters their chemical, physical, and morphological features in an uncontrollable way [125]. On the contrary, artificial systems used for drug delivery can be produced in a very controlled way [126]. The actual toxicity of micro- and nanoparticles for drug delivery should be investigated in great detail before their commercialization and daily use. In this context, a very big difference can be found in different categories of products. This difference is mostly due to their classification, which makes them enter into different regulatory systems [127]. This is particularly relevant in the case of preparations for pharmaceutical and cosmetic use. Plastic microparticles have been used for a long time in cosmetics as scrubbers or rheological modifiers or for the controlled release of active ingredients (e.g., anti-aging principles) [128]. The use of polymers in cosmetics has been limited by companies, mostly considering the recent concern of customers for the dispersion of micro- and nanoplastics in the environment and hence mostly for marketing reasons. Regulation for cosmetics is surely much more permissive than for pharmaceutical products [129,130]. Nevertheless, from a purely physico-chemical point of view, it is very difficult to distinguish a topical formulation for pharmaceutical use from a cosmetic one. Concern about nanomaterials in cosmetics has led to a partial modification of the regulation by restricting their use. However, the main limitation is the specification of the presence of nanosized particles in the description of a product and, in particular, in the list of its ingredients [131]. Other products that, together with cosmetics, may contribute to the release of plastic micro- and nanoparticles on the skin are pieces of garments [125]. Polyester clothes are already known to be some of the most important sources of pollution by microplastics in the environment but the general ability of commercial fabrics to release micro- and nanoparticles to the skin has not been investigated in detail. Accordingly, we suggest the investigation of the role of nano- and micro-materials released by cosmetic products and textiles in facilitating the penetration of potentially dangerous molecules through the skin.

### 8.3. Nanoparticles for Biomedical Applications and Micro- and Nanoplastics in the near Future

As previously discussed in the earlier sections, the utilization of nanoparticles in the biomedical field is an evolving topic; its foundations were laid in the past, but its future remains difficult to predict. However, we anticipate that over the next 5–10 years, the interaction between humans and nanotechnology will become an increasingly hot topic, particularly in terms of political, regulatory, and scientific discussions. On the one hand, it is highly likely that the increased use of nanotechnologies, such as nanoparticles, in the biomedical field will lead to greater regulation, consequently resulting in more restrictions and a heightened awareness of the actual benefits of such products on human health.

Thus, we expect that the continuous and constant study of nanomaterials will lead to the realization of increasingly effective and efficient solutions; simultaneously, our understanding of the toxicology of these materials will deepen, enabling the study of their long-term effects, a challenge given their recent clinical application. Moving on to the other topic of this review, the consideration of microplastics and nanoplastics necessitates the evaluation of multiple factors. A growing societal, political, and scientific awareness of environmental sustainability, coupled with the depletion of fossil fuels, is driving a market shift towards products with reduced fossil-based plastic content. This trend is reflected in the scientific community and industry, which are increasingly exploring the substitution of conventional plastics with biodegradable and renewable alternatives. Indeed, the utilization of rapidly biodegradable plastics would substantially decrease their environmental concentrations, thereby minimizing exposure of living organisms. That said, the potential toxicity of micro- and nano-bioplastics during the time interval between their formation and their degradation, and possibly of such degradation products, should not be underestimated.

Another alternative to conventional polymers is the use of lipid-based nanoparticles. Using these materials, such as liposomes, means having components that are already present in the human body. Further exploration of these systems as drug carriers and their skin penetration is required to have a proper design and enlarge the use of these materials.

The interconnectivity of nanomaterial use in biomedicine and nanomaterial toxicity, both of which involve the cutaneous penetration of micro- and nanomaterials, is evident. Regardless of the biological outcome, this shared mechanism highlights the imperative for interdisciplinary research. We envision a future where such an approach becomes the norm.

## 9. Conclusions

Skin is a potentially valuable target for the controlled delivery of diagnostic or therapeutic agents, offering some advantages over other ways of subministration, but, at the same time, the penetration of unwanted substances and pollutants can be extremely dangerous for human health. Recently, it has been demonstrated that, if properly designed, micro- and, even more efficiently, nanoparticles are able not only to pass the skin but also to enhance the transport of small molecules, features that distinguish them from traditional macroscopic materials. If, on the one side, this offers new opportunities in the field of nanomedicine, on the other side, it creates concern about the effects of micro- and nanoplastic pollutants. The possible toxicity of these particles is not completely understood yet but, besides this primary toxicity, a secondary toxicity given by the potential transport of molecular agents needs to be considered. In this paper, we have highlighted that the controlled delivery of drugs and unwanted transport of pollutants are strongly related and that they both depend on the natures, sizes, and shapes of the relevant particles. Moreover, the interaction of these materials with the skin is the crucial point to evaluate both their use as nanocarriers and their potential toxicity. The penetration of particles though the skin represents both an environmental concern and a promise for nanomedicine, and experimental results acquired in these two distinct fields should be merged to reach a deeper understanding of the phenomenon.

## Figures and Tables

**Table 1:** This table summarizes the chemical-–physical characteristics of the micro- and nanoparticles we took in consideration in the last 2 sections and their interaction with skin sections and cells, epidermis (EP), hair follicles (HFs), Stratum Corneum (SC), dermis (DE), dermal fibroblasts (DFs), and epidermal keratinocytes (EKs).

Material	Size (nm)	Shape	Z-Potential (mV)	Functionalization	Accumulation Region	Reference
PLGA	50 226	Sphere	−19.1 ± 2 −20 ± 1	_ _	EP HFs	[88]
PLGA	293 494	Sphere	−17.2 ± 0.6 −9.2 ± 0.2	_ _	SC, HFs SC	[89]
PLGA	50 100	Sphere	_ _	_ _	HFs SC	[90]
PLGA	30 30 30	Sphere	−46.1 ± 10 −19.3 ± 2 −4.5 ± 0.3	_ PEG3000 PEG1000	SC and HFs SC, HFs, EP, DE SC, HFs, EP, DE	[91]
PLGA Lichitin/ chitosan	281 275	Sphere	−5.63 ± 0.2 40.8 ± 2	_ _	HFs, DE EP	[92]
PLA PCL	50 32	Sphere	_ _	PEG PEG	SC, HFs, EP, DE SC	[93]
PS	200–6000	Sphere	_	_	The microspheres were internalized by DFs and EKs. Microplastics with sizes between 1000 and 2000 nm demonstrated the major uptake.	[103]
PS	50, 500, 1000	Sphere	_	_	The microspheres were internalized by DFs. Bigger microplastics demonstrated higher cytotoxicity.	[104]
PS	500<, 1000	Sphere	_	_	The microspheres were internalized by DFs. Smaller microplastics were internalized by cells; bigger microplastics remained in the cell walls.	[105]
PS	2000< 1000<	Irregular fragments	_ _	_ _	The microplastics penetrated into a 3D human skin model. Microplastics penetrated into EP and DE in both human and rat skin.	[106]
PS	5000–200,000	Irregular fragments	_	_	The microspheres were internalized by DFs. Upon increasing the roughness of microplastics, the researchers noticed an increase in cytotoxicity.	[107]
PS	100 100 100	Sphere	_ _ _	_ -COOH groups -NH_2_ groups	Microplastics disrupted DF wall cells. Functionalized microplastics disrupted the cell walls more, especially the one functionalized with -NH_2_ groups.	[108]

## Data Availability

No new data were created or analyzed in this study.
